# The functional insight into the genetics of cardiovascular disease: results from the post-GWAS study

**DOI:** 10.18699/VJGB-22-10

**Published:** 2022-02

**Authors:** L.O. Bryzgalov, E.E. Korbolina, I.S. Damarov, T.I. Merkulova

**Affiliations:** Institute of Cytology and Genetics of the Siberian Branch of the Russian Academy of Sciences, Novosibirsk, Russia; Institute of Cytology and Genetics of the Siberian Branch of the Russian Academy of Sciences, Novosibirsk, Russia; Institute of Cytology and Genetics of the Siberian Branch of the Russian Academy of Sciences, Novosibirsk, Russia; Institute of Cytology and Genetics of the Siberian Branch of the Russian Academy of Sciences, Novosibirsk, Russia; Novosibirsk State University, Novosibirsk, Russia

**Keywords:** non-coding genetic variation, rSNPs, cardio-vascular disease risk, GWAS association, 1000 Genomes Project, gene expression regulation, transcription factor binding, некодирующие полиморфизмы, регуляторные SNP, предрасположенность к сердечно-сосудистым заболеваниям, полногеномные исследования ассоциаций, проект «1000 геномов», регуляция экспрессии генов, транскрипционные факторы

## Abstract

Cardiovascular diseases (CVDs), the leading cause of death worldwide, generally refer to a range of pathological conditions with the involvement of the heart and the blood vessels. A sizable fraction of the susceptibility loci is known, but the underlying mechanisms have been established only for a small proportion. Therefore, there is an increasing need to explore the functional relevance of trait-associated variants and, moreover, to search for novel risk genetic variation. We have reported the bioinformatic approach allowing effective identif ication of functional non-coding variants by integrated analysis of genome-wide data. Here, the analysis of 1361 previously identif ied regulatory SNPs (rSNPs) was performed to provide new insights into cardiovascular risk. We found 773,471 coding co-segregating markers for input rSNPs using the 1000 Genomes Project. The intersection of GWAS-derived SNPs with a relevance to cardiovascular traits with these markers was analyzed within a window of 10 Kbp. The effects on the transcription factor (TF) binding sites were explored by DeFine models. Functional pathway enrichment and protein–protein interaction (PPI) network analyses were performed on the targets and the extended genes by STRING and DAVID. Eighteen rSNPs were functionally linked to cardiovascular risk. A signif icant impact on binding sites of thirteen TFs including those involved in blood cells formation, hematopoiesis, macrophage function, inf lammation, and vasoconstriction was found in K562 cells. 21 rSNP gene targets and 5 partners predicted by PPI were enriched for spliceosome and endocytosis KEGG pathways, endosome sorting complex and mRNA splicing REACTOME pathways. Related Gene Ontology terms included mRNA splicing and processing, endosome transport and protein catabolic processes. Together, the f indings provide further insight into the biological basis of CVDs and highlight the importance of the precise regulation of splicing and alternative splicing

## Abstract (ru)

Сердечно-сосудистые заболевания (ССЗ) занимают одно из ведущих мест по уровню смертности в развитых странах. На сегодняшний день значительное число геномных локусов продемонстрировали ассоциации с ССЗ, в основном в полногеномных исследованиях (GWAS), но лишь в немногих случаях установлены молекулярно-генетические механизмы, лежащие в основе ассоциации. Таким образом, задача определения функционально значимых для предрасположенности к ССЗ генетических вариантов остается актуальной. Ранее мы разработали биоинформатический подход, позволяющий эффективно идентифицировать регуляторные SNP (rSNP) – функциональные однонуклеотидные полиморфизмы, влияющие на экспрессию генов, путем анализа комплекса полногеномных данных. В данной работе с использованием данных проекта «1000 геномов» найден 773 471 кодирующий SNP, являющийся косегрегирующим маркером для 1361 идентифицированного нами rSNP. Проанализировано расположение этих маркеров в пределах геномного окна размером 10 Кб вокруг маркеров GWAS, ассоциированных с риском развития ССЗ или потенциально связанными с этим риском фенотипическими признаками. Влияние rSNP на сайты связывания транскрипционных факторов исследовано с помощью моделей DeFine. Анализ межбелковых взаимодействий и обогащения биологических путей для соответствующих генов-мишеней проведен с использованием баз STRING и DAVID. Найдено восемнадцать rSNP, функционально связанных с риском развития ССЗ. В клеточной линии K562 выявлено значительное влияние этих rSNP на сайты связывания тринадцати транскрипционных факторов, в том числе участвующих в процессах кроветворения, функционирования макрофагов, воспаления и вазоконстрикции. Для двадцати одного соответствующего гена- мишени, а также пяти функционально взаимодействующих с ними генов показано обогащение биологически- ми категориями сплайсосомы и эндоцитоза (KEGG), комплекса сортировки эндосом и процессов сплайсинга (REACTOME), а также некоторыми терминами генетических онтологий, в том числе сплайсинга и процессинга мРНК. В целом полученные результаты расширяют имеющиеся представления о роли rSNP и связанных с ними изменений экспрессии генов в развитии ССЗ и подчеркивают важность точной регуляции процессов сплайсин- га мРНК и альтернативного сплайсинга для формирования определенного фенотипа

## Introduction

While the running data indicate that the prevalence of cardiovascular disease may vary among regions of the world, they remain one of the leading causes of death and health loss and a large proportion of their forms are shown to have a familial aggregation and high heritability (Smith J.G., Newton-Cheh, 2015; Roth et al., 2017; Wang Y., Wang J.-G., 2018). The previous efforts led to the identification of candidate risk genes including the genes of renal homeostasis for Mendelian forms of abnormal blood pressure levels and several transcription factors (including NKX25, GATA4, TBX) for congenital septal (Kathiresan, Srivastava, 2012). However, the broad group of cardiovascular traits such as myocardial infarction/ischemia or coronary artery disease (CAD) show complex inheritance patterns, which suggest the collective and non-linear effects from multiple genetic and non-genetic factors. With the recent technological advances, the whole-exome sequencing (Li A.H. et al., 2017; Seidelmann et al., 2017; Khera et al., 2019) and genome-wide association studies, GWASs (in particular (Erdmann et al., 2018; Schunkert et al., 2018)) have been shown to be a powerful tool for discovering the genetic variation associated with cardiovascular risk. The outcomes from multiple GWASs and their meta-analysis completed during the past decade have been deposited in the catalogs, such as the catalog of published GWASs from The National Human Genome Research Institute (Buniello et al., 2019), the Coronary ARtery DIsease Genome-wide Replication And Meta-analysis, CARDIoGRAM (Preuss et al., 2010) plus The Coronary Artery Disease (C4D) Consortium and UK Biobank (Ge et al., 2017).

The genes related to regulating blood pressure, the tone and elasticity of the vascular wall, the inflammation process, the proliferation of vascular smooth muscle cells and the levels of low density lipoprotein cholesterol (LDC-C) are ‘traditionally’ involved in cardiovascular risk. Moreover, the GWA studies resulted in numerous loci for cardiometabolic risk factors such as plasma biomarkers of lipid metabolism, thrombosis, inflammation and metabolic status playing a role in risk analysis. In the case of long QT syndrome, in particular, fifteen candidate genes have been reported to date, including several genes for ion channels (Refsgaard et al., 2012; Arking et al., 2014). Notably, three candidates (KCNQ1, KCNH2, and SCN5A) account for approximately 75 % of cases (Wallace et al., 2019). And there are more than 150 suggestive loci estimated for CAD although only 46 from them have reached the genome-wide significance threshold (den Hoed et al., 2015). Interestingly, a considerable overlap has been shown between the risk genes for monogenic forms of CVDs and those generating an association signal in GWAS (Rau et al., 2015).

Together, these findings have developed a relatively comprehensible picture of the biology underlying the cardiovascular disease, but despite the advances, we are not able to analyze the functionality for the majority of the reported associated genetic loci. Among the reasons, there may be important limitation of GWAS for identifying risk genomic regions instead of risk genes and the non-coding localization of the majority of the susceptibility SNPs (Ward, Kellis, 2012). Current theories assume that so-called regulatory non-coding SNPs (rSNPs) seem to make the greatest contribution to the development of various multifactorial diseases including oncological and CAD as these are directly involved in the control of the gene expression levels.

One of the lessons learned is the growing need for the comprehensive post-GWAS analysis in order to translate the reported statistical association to uncover the causal variants amongst those in linkage disequilibrium (Mansur et al., 2018; Smith A.J.P. et al., 2018). Moreover, only some of GWASimplicated loci (i. e. sixteen of 46 validated loci for CAD) are also associated with ‘classic’ genes of risk, therefore showing the potential involvement of ‘non-traditional’ biological pathways in the disease (Smith J.G., Newton-Cheh, 2015).

Since the advances in next-generation sequencing technologies have provided an expanding amount of large-scale -omics datasets, the research strategies have started to focus on the integration of various genome-wide information layers (Huang S. et al., 2017) using functional genomics assays. One way to find putative functional variants is to detect regions with allele-specific binding of transcription factors or histone modifications, suggesting their different regulatory downstream role. ChIP-seq data will provide the snapshots of protein-DNA interactions allowing the analysis of sites with significant difference in signal between the alleles or allelic differences. The employment of transcriptome (RNA-seq) data will provide the snapshots of gene expression levels depending on the allele. The epigenome (iTEA) analysis (Meng et al., 2018) in combination with the regulatory sequence annotations, i. e. DNase-seq and ChIP-seq datasets (Cavalli et al., 2019), is beginning to be used to screen for the causal variants changing gene expression, including within GWAS-derived loci. However, the researchers have not come close to solving the issue of identifying the rSNPs at the genome-wide scale. The limitations are imposed by the incomplete experimental data collected to date and some critical methodological problems. Notably, one of the major challenges has become the development of effective in silico (bioinformatic) approaches but these are relatively few in number to date and only the individual studies have been reported to elucidate the underlying mechanisms of CVDs (Gong et al., 2018; Roman, Mohlke, 2018).

To address the challenges, we have recently reported an effective bioinformatic approach that facilitates the systematic identification of functional non-coding variants from available genome-wide data (Korbolina et al., 2018). Our pipeline utilized multiple positional and functional criteria to reveal non-coding regulatory variants in the human genome and imputed curated GWAS association signals to select the potentially colorectal cancer-causal rSNPs within a 1 Kb window of genomic sequence centered at the GWAS-SNP. Initially, the regulatory properties of found rSNPs were shown on a number of human cell lines of different origins (HCT116, K562, MCF7). However, expression of tissue-specific transcription factors is suppressed in cell lines. For this reason, here we have adopted the list of 1361 regulatory SNPs from the said study. The data from 1000 Genomes Project (1000 Genomes Project Consortium et al., 2015) were incorporated in the analysis to improve matching rSNPs with the phenotypic outcome that would be the risk of CVDs. Further, we tried to narrow the focus toward rSNPs that potentially result in a difference in predicted binding status of various transcription factors and performed the functional annotation of the targeted genes.

## Materials and methods

Input data on non-coding regulatory variants in human genome. Regulatory SNPs from our earlier study (Korbolina et al., 2018) were used for input including the data on identified targeted genes. All of these were associated with the allelespecific binding of various TFs and allele-specific expression from raw data.

Genetics data. Genetic variants and allele information were retrieved from dbSNP150 (Database resources…, 2016) and four 1000 Genomes Project super populations (AFR, AMR, ASN and EUR) (1000 Genomes Project Consortium et al., 2015). The GRCh37 annotation was used to map genetic variants to gene loci.

**Fig. 1.**
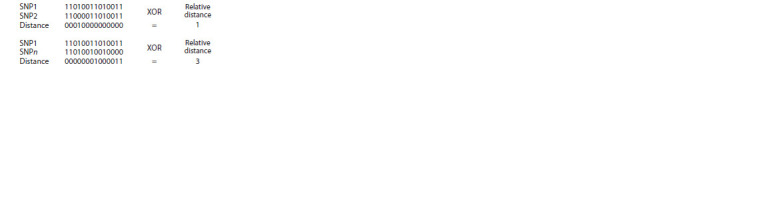
Finding the relative SNP-to-SNP distance using XOR. The binary code was used for genotypes (the symbols 0 and 1 were used to represent two different alleles in a heterozygous site).

Assessment of the SNP clustering with a distance measure. To map significant GWAS associations to novel functional variants here we implemented the data of 1000 Genomes Project. First, we extracted the data on 2500 individual haplotype-resolved human genomes from various super populations (AFR, AMR, ASN and EUR). We found 1361 variants from the 1476 input rSNPs reported earlier (Korbolina et al., 2018) within this genomic data. Next, we extracted the data on all SNPs within the transcribed genomic regions. The lists of rSNPs defined from 1000 genomes (‘population’ set of 1361 variant) and coding SNPs defined from 1000 genomes were consolidated. Each individual from 2500 genome samples was genotyped separately by each SNP from the consolidated list. The genotype data were turned to binary, where “0” represented the most frequent allele and “1” – the minor allele within the individual genotype. The resulting genotype data were set to the 2D matrix containing 2500 individual genomes and genotypes for all SNPs from the consolidated list. The initial matrix was then transformed to the distance matrix using XOR logic gate. Figure 1 shows an example of SNP distance measurement by XOR.

All the relative distances were normalized on the number of times the minor allele was found there in the genomic data of 2500 genomes. Based on the analysis of the resulting distances, we quantified the likelihood of the repeated recognition of rSNPs and the coding SNP within one genotype in human populations. Here we found that in total 773 471 coding SNPs ( p <0.01) may be merely co-segregating markers for our input rSNPs.

Implementing GWAS data to interpret the input rSNPs functionality. Next, we examined GWAS index SNPs available up to date (May 2019). We used the ‘cardio’ signature for querying the GWAS Catalog (including ‘heart’, ‘coronary artery disease’, ‘CAD’, ‘platelet’, ‘blood’, ‘blood cells’, ‘pressure’, ‘count’, ‘vessel’, ‘caliber’, ‘pulse’, ‘artery’). Next, we evaluated whether the input rSNPs or any of the corresponding coding markers lying within a 10 Kbp window of GWAS SNPs (Brodie et al., 2016) could be related to heart and vascular disease association signals (Suppl. Table 1).^1^


Supplementary Tables 1–4 are available in the online version of the paper: https://disk.icgbio.ru/s/zBiq4fm632zRywe 


A functional protein association network. STRING v 11 (Szklarczyk et al., 2019) was selected as the PPI database with a subset of 21 genes targeted by 18 rSNPs that were found to be associated with cardiovascular risk, as input (see Suppl. Table 2 for details of enrichment analysis).

Functional annotation by DAVID. DAVID Database for Annotation, Visualization and Integrated Discovery (Database resources…, 2016) was used to further interpret the same targeted genes with the default values set for all parameters. The outputs of DAVID functional annotation and clustering tools are given in the Suppl. Table 3.

In silico analysis of potentially affected TF binding sites. The sequence-based DeFine deep learning models were employed to predict the effects on transcription factor binding in K562 cells (the data are accessible online via the DeFine tool) and to rank 18 rSNPs identified for cardiovascular risk. The DeFine functional scores predict the transcriptional factor- DNA binding intensities and are appointed in the view of the differences between the reference sequence and the altered sequence, as reviewed in (Wang M. et al., 2018). The outputs including the maximum TF functional scores, the most likely candidate TFs and top 10 contact genes for each rSNP position are given in the Suppl. Table 4.

R code. The R package, version 3.1.0, was used for data analysis.The custom-made Perl scripts employed are available upon request.

## Results

Investigating rSNP functional relevance to cardiovascular risk via GWAS associations

We analyzed 438 GWAS-SNPs with relevance for cardiovascular traits and defined eighteen candidate rSNP variants at a 10 Kbp window size (see Suppl. Table 1) including within the associated loci for coronary heart and artery disease (CAD, 3 loci), HLD cholesterol, QT interval, red blood cells and platelet cells traits. One interesting result was that ten GWASderived SNPs including the ones for phenotypical associations with systolic and diastolic blood pressure, pulse pressure, retinal arteriolar microcirculation and one for CAD entered the list of founded regulatory SNPs in the study. We considered the input rSNP targets (Korbolina et al., 2018), and any gene targeted to these ten GWAS-SNPs (initially from GWAS catalog) to be a candidate for mediating the association. Actually, only one cardio-related SNP out of eighteen was linked to more than one target gene: rs3744061 (MFSD11, JMJD6).

Functional annotation of the rSNP targeted genes

**Fig. 2.**
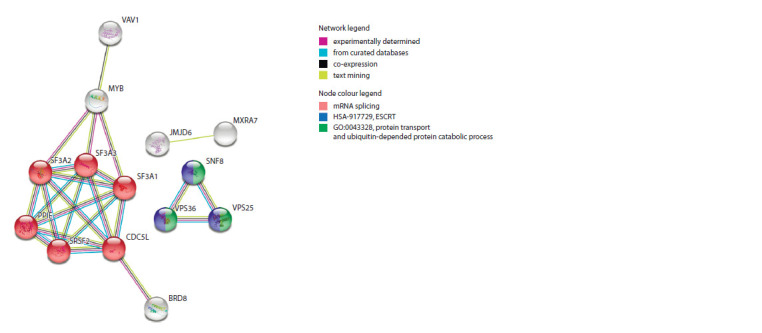
Protein association network for 21 rSNP-targeted genes and 5 gene partners with a functional relevance to cardiovascular risk in STRING. The network for rSNP targets has been expanded by additional 5 proteins (via the ‘More’ button in the STRING interface and default confidence cut-off). The network contains 26 nodes with 24 edges (vs 10 expected edges, the disconnected nodes are hidden); enrichment p-value < 0.001. The legend inset at the right shows the various types of evidence for the predicted association and the enriched annotation term for the protein (by different colours of the nodes).

We further looked into the target genes to these eighteen cardio-vascular risk rSNPs as candidates for mediating the effects on CVDs. In our STRING enrichment analysis (Szklarczyk et al., 2019), 21 rSNP targeted genes had five predicted partners and these were shown significantly enriched in Kyoto Encyclopedia of Genes and Genomes (KEGG) spliceosome pathway, two REACTOME pathways (endosomal sorting complex required for transport and mRNA splicing) and 40 Gene Ontology terms including gene expression, RNA splicing, regulation of mRNA splicing, regulation of alternative mRNA splicing via spliceosome, protein transport to vacuole involved in ubiquitin-dependent protein catabolic process via the multivesicular body sorting pathway; ubiquitindependent protein catabolic process (see Suppl. Table 2 for the details of STRING enrichment analysis). Figure 2 shows the corresponding protein association network with evidence for association between the targets. Such an enrichment indicates that the input proteins are at least partially biologically connected as a group.

The same enriched groups were found by DAVID functional annotation tool (Huang D.W. et al., 2008), including mRNA splicing and mRNA 3′-splice site recognition, regulation of transcription and endosomal transport GO terms (see Suppl. Table 3). However, these processes could all be linked to the pathological features of CVDs, but the most notable is the group of targets that are associated with splicing and alternative splicing regulation (given in red nodes).

DeFine rSNP prioritization

The DeFine online tool revealed that eight rSNPs had positive functional scores meeting the pathogenic potential to enhance TF binding, and five – to weaken TF binding (see Suppl. Table 4) in the supported K562 cell line. The binding sites of ten TFs were most strongly altered by 13 rSNPs according to DeFine models: TAL1, REST, NR1H2, SP2, RFX5, MXI1, PBX2, NFYB, ZNF274 and ZNF263. Nine out of ten of these TFs (with the exception of ZNF274) were the proteins with antibodies to which the immunoprecipitation was made by the original authors (as given in the Supplementary material, Korbolina et al., 2018). This would be expected in principle when examining certain regulatory regions.

In more detail, rs210962 (GWAS-derived) and rs7920217 functional variants shared the potential to weaken the binding of RE1 silencing transcription factor (REST) to the related genomic loci (the DeFine scores of –0.0778 and –0.0888, respectively). rs2270574 and rs8106212 within the GWAS loci for CAD and platelet trait, respectively, shared the association with SP2 transcription factor, but had the opposite effects on TF binding according to DeFine scores (0.05020 to enhance and –0.0973 to weaken the binding, respectively). And again, two rSNPs shared the effects on TF binding sites for T-cell acute leukemia protein 1 (TAL1) in this study: rs140492 within the locus for HDL cholesterol (DeFine score 0.0996 to enhance the TF binding was counted) and rs10445033 – within GWAS locus for red blood cell levels with the DeFine score –0.0891 to weaken the TF binding. Again, DeFine identified the same targeted genes (i. e. SNF8 for rs2270574) with additional potential targets in each rSNP case (ten top candidate genes are listed in the DeFine output data, see Suppl. Table 4).

## Discussion

As has been mentioned in the Results, we have identified eighteen rSNPs with a functional relevance to CVDs that matched the GWAS loci for coronary heart and artery disease, HLD cholesterol, retinal arteriolar microcirculation, QT interval, red blood cells and platelet cells traits from this study (see Suppl. Table 1). The genome position of rSNP coincided with that for GWAS-derived SNP for ten identified variants out of eighteen. It was an interesting find as this was a relatively large part compared to our previously reported results for colorectal cancer (Korbolina et al., 2018) and cognitive disorders (Bryzgalov et al., 2018).

As has been mentioned in the Results, we have identified eighteen rSNPs with a functional relevance to CVDs that matched the GWAS loci for coronary heart and artery disease, HLD cholesterol, retinal arteriolar microcirculation, QT interval, red blood cells and platelet cells traits from this study (see Suppl. Table 1). The genome position of rSNP coincided with that for GWAS-derived SNP for ten identified variants out of eighteen. It was an interesting find as this was a relatively large part compared to our previously reported results for colorectal cancer (Korbolina et al., 2018) and cognitive disorders (Bryzgalov et al., 2018).

What we should like to mention is that a number of truly critical processes depend on the blood cells functionality and biological activities as has been widely demonstrated. Regarding erythrocytes, these include not only oxygen transport, but immune response (Astle et al., 2016), redox homeostasis (Kuhn et al., 2017) and regulation of vascular function (Helms et al., 2018; Rifkind et al., 2018). Moreover, several ex vivo studies on diabetes mellitus identified that red blood cells do act to mediate the development of endothelial dysfunction and cardiac injury (Yang et al., 2013; Zhou et al., 2018; Pernow et al., 2019). This means that any qualitative or quantita-tive deviations from the physiological ranges may be closely linked to the disease (Leal et al., 2018). The data suggest that the blood cell count and hematological parameters could be useful markers to improve the cardiovascular risk prediction; however, they have limited sensitivity (Mozos, 2015; Samman Tahhan et al., 2017; Lassale et al., 2018; Haybar et al., 2019). Interestingly, it was shown that the level of expression of some curated genes may independently aid in the prediction of heart failure prognosis when combined with neutrophilto- lymphocyte ratio (Wan et al., 2018). The risk for CVDs correlates well with platelet traits (Sloan et al., 2015; Vélez, García, 2015; Reinthaler et al., 2016; Gill et al., 2018), the initiation and progression of CAD in particular (Uysal et al., 2016). However, some have argued that shared genetic pathways linking blood cells with complex pathologies, including autoimmune diseases, schizophrenia, and CAD may be noncausal (Astle et al., 2016).

Still, our current knowledge of splicing regulation and alternative splicing in the heart is limited, but splicing analysis has emerged as an important line of research for the cardiovascular risk. The studies revealed that the regulation of splicing and alternative splicing events (reviewed in (van den Hoogenhof et al., 2016)) seem to play a causative role in heart development and cardiovascular disease. The promising therapeutic targets (Rexiati et al., 2018) have already been proposed. There is evidence that a significant number of alternate transcripts are increased in diseased hearts compared to controls, and can be involved in disease. Thus, abnormal splicing of apoptotic genes contributes to the pathogenesis of several CVDs including dilated and diabetic cardiomyopathy, atherosclerosis and heart failure as reviewed in (Dlamini et al., 2015). The dysregulation of cardiac splicing factors can also be sufficient to affect heart function and lead to disease. Thus, there is evidence that the decrease in RNA-binding motif protein 20 (RBM20) levels may be involved in dilated cardiomyopathy by providing input to splicing of at least several known target genes (Maatz et al., 2014). In the study, the target gene of the rs4360494 functional variant within the GWAS-derived locus for pulse pressure is the SF3A3 gene that encodes subunit 3 of the splicing factor 3a protein heterotrimeric complex. As is known, the splicing factor 3a plays an important role in U2 snRNP biogenesis and thus, pre-mRNA splicing (Krämer et al., 2005; Huang C.-J. et al., 2011). With respect to other pathological states, targeting the components of the spliceosome has full potential as a strategy for cancer treatment and prognosis (Lin, 2017; El Marabti, Younis, 2018; Martinez-Montiel et al., 2018). Thus, the data suggest that SF3A3 is involved in the p53 activation, the induction of cell cycle arrest and cell death in non-small cell lung cancer (Siebring-van Olst et al., 2017). The SRSF2 gene, encoding another splicing machinery component, could be used as a reliable prognostic factor in patients with hepatocellular carcinoma (Luo et al., 2017).

The role of endosomal system in the heart functioning and cardiovascular disease is described as critical, too (Yang et al., 2019), as endosomes contribute to control of cholesterol (LDL) plasma levels (Wijers et al., 2018), Ca2+ homeostasis and protein trafficking (Curran et al., 2015) and play an important part in atherosclerosis risk (Cai et al., 2018). However, surprisingly little is known regarding the regulation of endosome- based protein trafficking in the heart. Thus, our results could serve as useful background for further research. Protein quality and control and ubiquitin-proteasome system, UPS (Gilda et al., 2016; Barac et al., 2017; Gilda, Gomes, 2017; Dorsch et al., 2019; Shukla, Rafiq, 2019) have also played an essential role in the initiation and progression of CVDs. In short, the findings suggest that UPS contributes to structural remodeling of the myocardium, ischemia-reperfusion injury and myocardial cell loss, important components of progressive heart failure. There is evidence for the non-degradative role as well, as the ubiquitination was shown to affect the important regulators of signaling pathways including those for the cell growth and apoptosis, DNA damage response, the innate immune response, endocytosis, and protein activity (reviewed partly in (Gupta et al., 2018)). Given that proteostasis is a dynamic multiple-step process involving complex molecular machinery, the deregulation at any stage could therefore be implicated in a wide variety of outcomes

When addressing directly the list of targets for our 18 rSNPs that are likely relevant to cardiovascular function, a member of the VAV gene family – VAV1 is the target of the rs8106212 variant for platelet distribution. Overall, the VAV proteins are known to interact with the receptors on cell surface to activate various downstream biological pathways thus leading to the alterations of transcription. VAV1 is important in hematopoiesis, playing a role in T-cell and B-cell development and activation (Rodríguez-Fdez, Bustelo, 2019). Another candidate, the PIEZO1 gene targeted by rs10445033 is widely associated with hereditary human diseases (Alper, 2017) and tissue homeostasis (Zhong et al., 2018). The homotetramer of the PIEZO1 protein functions as a pore-forming subunit of a mechanically activated cation channel and contributes a lot to vascular biology and development (Li J. et al., 2014) including mechanistic signaling in endothelium (Albarrán-Juárez et al., 2018). Some studies suggest that the molecular events involved in the development of acute myocardial infarction may include MiR-103a microRNA expression in plasma and the subsequent regulation of the expression of PIEZO1 protein (Huang L. et al., 2013). However, the up-regulation of Piezo1 was demonstrated in rat model of heart failure (Liang et al., 2017).

Since the rSNPs do induce the variation in the gene expression, the significant rSNPs’ effects on the binding affinity of a genomic locus for transcription factors can be a cause (Deplancke et al., 2016). There is relative evidence for the GWAS loci for complex diseases to be associated with not only ultimate changes in gene expression (Gallagher, Chen-Plotkin, 2018) but with the activity of various TFs (Harley et al., 2018). Here, we used the online classifier of variant pathogenicity, DeFine (Wang M. et al., 2018), to explore the functional effects of 18 rSNPs with the relevance to cardiovascular traits on the TF binding sites. DeFine classification approach employs the sequence-based deep learning models between the reference sequence and the altered sequence centered at the variant. The authors have shown that the given tool is capable to identify the causal non-coding variants within the reported GWAS loci for complex human diseases.

In this study, a significant functional impact on binding sites of thirteen TFs, including five genomic positions with potentially weakened and eight – with potentially enhanced TF binding, was found in K562 cells. Among ten identified TFs with maximum DeFine functional scores (see Suppl. Table 4), four TFs (NFYB, PBX2, SP2 and TAL1) were functionally reliable to CVDs when considering well-known roles. Thus, TAL1 is the erythroid differentiation factor that cooperates with various TFs to regulate hematopoiesis and normal differentiation of myeloid cells, and may also contribute to the process of malignant transformation (Vagapova et al., 2018). PBX genes encode homeodomain transcription factors, that were shown to determine the allele-specific phenotypic presentation of heart defects in mice and their loss resulted in the insufficient expression of both genes controlling the blood vessel widening and narrowing and finally led to persistent vasoconstriction by multiple pathways (McCulley et al., 2017). NFYB (nuclear transcription factor Y subunit beta) is a subunit of a highly conserved trimeric TF that binds with high specificity to CCAAT motifs in the promoter regions in a variety of genes. Interestingly, the evidence for Nf-y, SP family factors (that bind to GC-boxes) and PBX1 to cooperate was identified (Suske, 2017; Völkel et al., 2018).

It is important that the regulatory elements of genome, the distribution of TF binding sites, and the effects of rSNPs on the gene expression can be highly tissue or cell line specific (Zhang et al., 2018). But since the tissue samples, in particular of the brain and heart, are very difficult to obtain from humans, it is not surprising that the approaches to genome-wide identifying of the functional variants were trained and available in cells and animal models first. It can be argued that genomewide studying of the functional effects of non-coding variants on transcription very often relies on the modeling of the cell type-specific binding of transcription factors to regulatory elements of genome. The interest in the field of reliable in silico methods is increasing, but there are only a few that have been more or less broadly implemented according to PubMed analysis (Wang M. et al., 2018). Moreover, the evidence suggests that the performance of different functional prediction tools varies by disease phenotype (Anderson, Lassmann, 2018) and thus may give contradictory statements.

Overall, to date, using GWAS associations seems the most common way to explore the non-coding variants in the terms of functionality. Still, a survey from published studies showed that this approach helps to interpret just a minor part of thousands of identified rSNPs (Cavalli et al., 2016, 2019). Our results suggest that the reported analysis pipeline integrating the datasets from 1000 Genomes Project may serve as a general framework for future research and would eventually lead to investigation of novel functional variants within significant GWAS loci that confer human disease risk. Considering our previously obtained data for colorectal cancer (Korbolina et al., 2018) and a number of cognitive disorders (Bryzgalov et al., 2018), we have more evidence for the precise regulation of splicing mechanisms and alternative splicing to be among the key mechanisms underlying the effects of non-coding genetic variation on the phenotype including various pathological conditions.

## Conclusion

Overall using GWAS associations seems the most commonly used way to explore the non-coding variants in the terms of functionality to date. Still, a survey from published studies showed that this approach helps to interpret just a minor part of thousands of identified rSNPs (Cavalli et al., 2016, 2019). Our results suggest that the reported analysis pipeline integrating the datasets from 1000 Genomes Project may serve as a general framework for future research and would eventually lead to investigation of novel functional variants within significant GWAS loci that confer human disease risk. In consideration of our previously obtained data for colorectal cancer (Korbolina et al., 2018) and a number of cognitive disorders (Bryzgalov et al., 2018), we have got another evidence for the precise regulation of splicing mechanisms and alternative splicing to be among the key mechanisms underlying the effects of noncoding genetic variation on the phenotype including various pathological conditions.

## Conflict of interest

The authors declare no conflict of interest.

## References

1000 Genomes Project Consortium, Auton A., Brooks L.D., Durbin
R.M., Garrison E.P., Kang H.M., Korbel J.O., Marchini J.L.,
McCarthy S., McVean G.A., Abecasis G.R. A global reference for
human genetic variation. Nature. 2015;526:68-74. DOI 10.1038/
nature15393.

Albarrán-Juárez J., Iring A., Wang S., Joseph S., Grimm M., Strilic B.,
Wettschureck N., Althoff T.F., Offermanns S. Piezo1 and Gq/G11
promote
endothelial inflammation depending on flow pattern and
integrin activation. J. Exp. Med. 2018;215(10):2655-2672. DOI
10.1084/jem.20180483

Alper S.L. Genetic Diseases of PIEZO1 and PIEZO2 Dysfunction. Curr.
Top. Membr. 2017;79:97-134. DOI 10.1016/bs.ctm.2017.01.001

Anderson D., Lassmann T. A phenotype centric benchmark of variant
prioritisation tools. NPJ Genom. Med. 2018;3:5. DOI 10.1038/
s41525-018-0044-9.

Arking D.E., Pulit S.L., Crotti L., van der Harst P., Munroe P.B., Koopmann
T.T., Sotoodehnia N., Rossin E.J., Morley M., Wang X., …
Schwartz P.J., Kääb S., Chakravarti A., Ackerman M.J., Pfeufer A.,
de Bakker P.I.W., Newton-Cheh C. Genetic association study of
QT interval highlights role for calcium signaling pathways in
myocardial repolarization. Nat. Genet. 2014;46(8):826-836. DOI
10.1038/ng.3014.

Astle W.J., Elding H., Jiang T., Allen D., Ruklisa D., Mann A.L.,
Mead D., Bouman H., Riveros-Mckay F., Kostadima M.A., …
Bourque G., Frontini M., Danesh J., Roberts D.J., Ouwehand W.H.,
Butterworth A.S., Soranzo N. The allelic landscape of human blood
cell trait variation and links to common complex disease. Cell. 2016;
167(5):1415-1429.e19. DOI 10.1016/j.cell.2016.10.042.

Barac Y.D., Emrich F., Krutzwakd-Josefson E., Schrepfer S., Sampaio
L.C., Willerson J.T., Robbins R.C., Ciechanover A., Mohr F.- W.,
Aravot D., Taylor D.A. The ubiquitin-proteasome system: a potential
therapeutic target for heart failure. J. Heart Lung Transplant.
2017;36(7):708-714. DOI 10.1016/j.healun.2017.02.012.

Brodie A., Azaria J.R., Ofran Y. How far from the SNP may the causative
genes be? Nucleic Acids Res. 2016;44(13):6046-6054. DOI
10.1093/nar/gkw500.

Bryzgalov L.O., Korbolina E.E., Brusentsov I.I., Leberfarb E.Y., Bondar
N.P., Merkulova T.I. Novel functional variants at the GWASimplicated
loci might confer risk to major depressive disorder, bipolar
affective disorder and schizophrenia. BMC Neurosci. 2018;
19(Suppl.1):22. DOI 10.1186/s12868-018-0414-3.

Buniello A., MacArthur J.A.L., Cerezo M., Harris L.W., Hayhurst J.,
Malangone C., McMahon A., Morales J., Mountjoy E., Sollis E.,
Suveges D., Vrousgou O., Whetzel P.L., Amode R., Guillen J.A.,
Riat H.S., Trevanion S.J., Hall P., Junkins H., Flicek P., Burdett T.,
Hindorff L.A., Cunningham F., Parkinson H. The NHGRI-EBI
GWAS Catalog of published genome-wide association studies, targeted
arrays and summary statistics. Nucleic Acids Res. 2019;47(1):
1005-1012. DOI 10.1093/nar/gky1120.

Cai Y., Wang X.-L., Flores A.M., Lin T., Guzman R.J. Inhibition of endo-
lysosomal function exacerbates vascular calcification. Sci. Rep.
2018;8(1):3377. DOI 10.1038/s41598-017-17540-6.

Cavalli M., Baltzer N., Pan G., Bárcenas Walls J.R., Smolinska Garbulowska
K., Kumar C., Skrtic S., Komorowski J., Wadelius C.
Studies of liver tissue identify functional gene regulatory elements
associated
to gene expression, type 2 diabetes, and other metabolic
diseases. Hum. Genomics. 2019;13(1):20. DOI 10.1186/s40246-019-
0204-8.

Cavalli M., Pan G., Nord H., Wallén Arzt E., Wallerman O., Wadelius
C. Allele-specific transcription factor binding in liver and cervix
cells unveils many likely drivers of GWAS signals. Genomics. 2016;
107(6):248-254. DOI 10.1016/j.ygeno.2016.04.006.

Curran J., Makara M.A., Mohler P.J. Endosome-based protein trafficking
and Ca2+ homeostasis in the heart. Front. Physiol. 2015;6:34.
DOI 10.3389/fphys.2015.00034.

Database resources of the National Center for Biotechnology Information.
Nucleic Acids Res. 2016;44(D1):D7-D19. DOI 10.1093/nar/
gkv1290

den Hoed M., Strawbridge R.J., Almgren P., Gustafsson S., Axelsson
T., Engström G., de Faire U., Hedblad B., Humphries S.E.,
Lindgren C.M., Morris A.P., Östling G., Syvänen A.-C., Tremoli E.,
Hamsten A., Ingelsson E., Melander O., Lind L. GWAS-identified
loci for coronary heart disease are associated with intima-media
thickness and plaque presence at the carotid artery bulb. Atherosclerosis.
2015;239(2):304-310. DOI 10.1016/j.atherosclerosis.2015.
01.032.

Deplancke B., Alpern D., Gardeux V. The genetics of transcription
factor DNA binding variation. Cell. 2016;166(3):538-554. DOI
10.1016/j.cell.2016.07.012.

Dlamini Z., Tshidino S., Hull R. Abnormalities in alternative splicing
of apoptotic genes and cardiovascular diseases. Int. J. Mol. Sci.
2015;16(11):27171-27190. DOI 10.3390/ijms161126017

Dorsch L.M., Schuldt M., Knežević D., Wiersma M., Kuster D.W.D.,
van der Velden J., Brundel B.J.J.M. Untying the knot: protein quality
control in inherited cardiomyopathies. Pflug. Arch. Eur. J. Physiol.
2019;471(5):795-806. DOI 10.1007/s00424-018-2194-0.

El Marabti E., Younis I. The cancer spliceome: reprograming of alternative
splicing in cancer. Front. Mol. Biosci. 2018;5:80. DOI 10.3389/
fmolb.2018.00080

Erdmann J., Kessler T., Munoz Venegas L., Schunkert H. A decade of
genome-wide association studies for coronary artery disease: the
challenges ahead. Cardiovasc. Res. 2018;114(9):1241-1257. DOI
10.1093/cvr/cvy084.

Gallagher M.D., Chen-Plotkin A.S. The post-GWAS era: from association
to function. Am. J. Hum. Genet. 2018;102(5):717-730. DOI
10.1016/j.ajhg.2018.04.002.

Ge T., Chen C.-Y., Neale B.M., Sabuncu M.R., Smoller J.W. Phenomewide
heritability analysis of the UK Biobank. PLoS Genet. 2017;
13(4):e1006711. DOI 10.1371/journal.pgen.1006711.

Gilda J.E., Gomes A.V. Proteasome dysfunction in cardiomyopathies.
J. Physiol. 2017;595(12):4051-4071. DOI 10.1113/JP273607.

Gilda J.E., Lai X., Witzmann F.A., Gomes A.V. Delineation of molecular
pathways involved in cardiomyopathies caused by troponin
T mutations. Mol. Cell. Proteom. 2016;15(6):1962-1981. DOI
10.1074/mcp.M115.057380.

Gill D., Monori G., Georgakis M.K., Tzoulaki I., Laffan M. Genetically
determined platelet count and risk of cardiovascular disease. Arterioscler.
Thromb. Vasc. Biol. 2018;38(12):2862-2869. DOI 10.1161/
ATVBAHA.118.311804.

Gong J., Qiu C., Huang D., Zhang Y., Yu S., Zeng C. Integrative functional
analysis of super enhancer SNPs for coronary artery disease.
J. Hum. Genet. 2018;63(5):627-638. DOI 10.1038/s10038-018-
0422-2.

Gupta I., Varshney N.K., Khan S. Emergence of members of TRAF
and DUB of ubiquitin proteasome system in the regulation of hypertrophic
cardiomyopathy. Front. Genet. 2018;9:336. DOI 10.3389/
fgene.2018.00336

Harley J.B., Chen X., Pujato M., Miller D., Maddox A., Forney C.,
Magnusen A.F., Lynch A., Chetal K., Yukawa M., Barski A., Salomonis
N., Kaufman K.M., Kottyan L.C., Weirauch M.T. Transcription
factors operate across disease loci, with EBNA2 implicated
in autoimmunity. Nat. Genet. 2018;50(5):699-707. DOI 10.1038/
s41588-018-0102-3.

Haybar H., Pezeshki S.M.S., Saki N. Evaluation of complete blood
count parameters in cardiovascular diseases: an early indicator of
prognosis? Exp. Mol. Pathol. 2019;110:104267. DOI 10.1016/
j.yexmp.2019.104267

Helms C.C., Gladwin M.T., Kim-Shapiro D.B. Erythrocytes and vascular
function: oxygen and nitric oxide. Front. Physiol. 2018;9:125.
DOI 10.3389/fphys.2018.00125.

Huang C.-J., Ferfoglia F., Raleff F., Krämer A. Interaction domains and
nuclear targeting signals in subunits of the U2 small nuclear ribonucleoprotein
particle-associated splicing factor SF3a. J. Biol. Chem.
2011;286(15):13106-13114. DOI 10.1074/jbc.M110.201491

Huang D.W., Sherman B.T., Lempicki R.A. Systematic and integrative
analysis of large gene lists using DAVID bioinformatics resources.
Nat. Protoc. 2008;4(1):44-57. DOI 10.1038/nprot.2008.211

Huang L., Li L., Chen X., Zhang H., Shi Z. MiR-103a targeting Piezo1
is involved in acute myocardial infarction through regulating endothelium
function. Cardiol. J. 2013;23(5):556-562. DOI 10.5603/
CJ.a2016.0056

Huang S., Chaudhary K., Garmire L.X. More is better: recent progress
in multi-omics data integration methods. Front. Genet. 2017;8:84.
DOI 10.3389/fgene.2017.00084

Kathiresan S., Srivastava D. Genetics of human cardiovascular disease.
Cell. 2012;148(6):1242-1257. DOI 10.1016/j.cell.2012.03.001.

Khera A.V., Chaffin M., Zekavat S.M., Collins R.L., Roselli C., Natarajan
P., Lichtman J.H., D’Onofrio G., Mattera J., Dreyer R., Spertus
J.A., Taylor K.D., Psaty B.M., Rich S.S., Post W., Gupta N.,
Gabriel S., Lander E., Ida Chen Y.-D., Talkowski M.E., Rotter
J.I., Krumholz H.M., Kathiresan S. Whole-genome sequencing
to characterize monogenic and polygenic contributions in patients
hospitalized with early-onset myocardial infarction. Circulation.
2019;139(13):1593-1602. DOI 10.1161/CIRCULATIONAHA.118.
035658.

Korbolina E.E., Brusentsov I.I., Bryzgalov L.O., Leberfarb E.Y., Degtyareva
A.O., Merkulova T.I. Novel approach to functional SNPs
discovery from genome-wide data reveals promising variants for
colon cancer risk. Hum. Mutat. 2018;39(6):851-859. DOI 10.1002/
humu.23425

Krämer A., Ferfoglia F., Huang C.-J., Mulhaupt F., Nesic D., Tanackovic
G. Structure–function analysis of the U2 snRNP-associated
splicing factor SF3a. Biochem. Soc. Trans. 2005;33(Pt.3):439-442.
DOI 10.1042/BST0330439

Kuhn V., Diederich L., Keller T.C.S., Kramer C.M., Lückstädt W., Panknin
C., Suvorava T., Isakson B.E., Kelm M., Cortese-Krott M.M.
Red blood cell function and dysfunction: redox regulation, nitric
oxide metabolism, anemia. Antioxid. Redox Signal. 2017;26(13):
718-742. DOI 10.1089/ars.2016.6954

Lassale C., Curtis A., Abete I., van der Schouw Y.T., Verschuren
W.M.M., Lu Y., Bueno-de-Mesquita H.B. Elements of the complete
blood count associated with cardiovascular disease incidence:
findings from the EPIC-NL cohort study. Sci. Rep. 2018;8(1):3290.
DOI 10.1038/s41598-018-21661-x.

Leal J.K.F., Adjobo-Hermans M.J.W., Bosman G.J.C.G.M. Red blood
cell homeostasis: mechanisms and effects of microvesicle generation
in health and disease. Front. Physiol. 2018;9:703. DOI 10.3389/
fphys.2018.00703.

Li A.H., Hanchard N.A., Furthner D., Fernbach S., Azamian M.,
Nicosia A., Rosenfeld J., Muzny D., D’Alessandro L.C.A., Morris
S., Jhangiani S., Parekh D.R., Franklin W.J., Lewin M., Towbin
J.A., Penny D.J., Fraser C.D., Martin J.F., Eng C., Lupski J.R.,
Gibbs R.A., Boerwinkle E., Belmont J.W. Whole exome sequencing
in 342 congenital cardiac left sided lesion cases reveals extensive genetic
heterogeneity and complex inheritance patterns. Genome Med.
2017;9(1):95. DOI 10.1186/s13073-017-0482-5.

Li J., Hou B., Tumova S., Muraki K., Bruns A., Ludlow M.J., Sedo A.,
Hyman A.J., McKeown L., Young R.S., Yuldasheva N.Y., Majeed Y.,
Wilson L.A., Rode B., Bailey M.A., Kim H.R., Fu Z., Carter D.A.L.,
Bilton J., Imrie H., Ajuh P., Dear T.N., Cubbon R.M., Kearney M.T.,
Prasad K.R., Evans P.C., Ainscough J.F.X., Beech D.J. Piezo1 integration
of vascular architecture with physiological force. Nature.
2014;515(7526):279-282. DOI 10.1038/nature13701.

Liang J., Huang B., Yuan G., Chen Y., Liang F., Zeng H., Zheng S.,
Cao L., Geng D., Zhou S. Stretch-activated channel Piezo1 is upregulated
in failure heart and cardiomyocyte stimulated by AngII.
Am. J. Transl. Res. 2017;9(6):2945-2955.

Lin J.-C. Therapeutic applications of targeted alternative splicing to
cancer treatment. Int. J. Mol. Sci. 2017;19(1):75. DOI 10.3390/
ijms19010075

Luo C., Cheng Y., Liu Y., Chen L., Liu L., Wei N., Xie Z., Wu W.,
Feng Y. SRSF2 regulates alternative splicing to drive hepatocellular
carcinoma development. Cancer Res. 2017;77(5):1168-1178. DOI
10.1158/0008-5472.CAN-16-1919.

Maatz H., Jens M., Liss M., Schafer S., Heinig M., Kirchner M., Adami
E., Rintisch C., Dauksaite V., Radke M.H., Selbach M., Barton
P.J.R., Cook S.A., Rajewsky N., Gotthardt M., Landthaler M.,
Hubner N. RNA-binding protein RBM20 represses splicing to orchestrate
cardiac pre-mRNA processing. J. Clin. Investig. 2014;
124(8):3419-3430. DOI 10.1172/JCI74523.

Mansur Y.A., Rojano E., Ranea J.A.G., Perkins J.R. Analyzing the effects
of genetic variation in noncoding genomic regions. In: Deigner
H.-P., Kohl M. (Eds.). Precision Medicine. Tools and Quantitative
Approaches. Acad. Press, 2018;119-144. DOI 10.1016/
B978-0-12-805364-5.00007-X.

Martinez-Montiel N., Rosas-Murrieta N., Anaya Ruiz M., Monjaraz-
Guzman E., Martinez-Contreras R. Alternative splicing as a target
for cancer treatment. Int. J. Mol. Sci. 2018;19:545. DOI 10.3390/
ijms19020545.

McCulley D.J., Wienhold M.D., Hines E.A., Hacker T.A., Rogers A.,
Pewowaruk R.J., Zewdu R., Chesler N.C., Selleri L., Sun X. PBX
transcription factors drive pulmonary vascular adaptation to birth.
J. Clin. Investig. 2017;128(2):655-667. DOI 10.1172/JCI93395

Meng F., Yuan G., Zhu X., Zhou Y., Wang D., Guo Y. Functional
variants identified efficiently through an integrated transcriptome
and epigenome analysis. Sci. Rep. 2018;8(1):2959. DOI 10.1038/
s41598-018-21024-6.

Mozos I. Mechanisms linking red blood cell disorders and cardiovascular
diseases. BioMed Res. Int. 2015;2015:682054. DOI 10.1155/
2015/682054.

Pernow J., Mahdi A., Yang J., Zhou Z. Red blood cell dysfunction:
a new player in cardiovascular disease. Cardiovasc. Res. 2019;
115(11):1596-1605. DOI 10.1093/cvr/cvz156.

Preuss M., König I.R., Thompson J.R., Erdmann J., Absher D., Assimes
T.L., Blankenberg S., Boerwinkle E., Chen L., Cupples L.A.,
Hall A.S., Halperin E., Hengstenberg C., Holm H., Laaksonen R.,
Li M., März W., McPherson R., Musunuru K., Nelson C.P., Burnett
M.S., Epstein S.E., O’Donnell C.J., Quertermous T., Rader
D.J.,
Roberts R., Schillert A., Stefansson K., Stewart A.F.R., Thorleifsson
G., Voight B.F., Wells G.A., Ziegler A., Kathiresan S., Reilly
M.P., Samani N.J., Schunkert H., and on behalf of the CARDIoGRAM
Consortium. Design of the Coronary ARtery DIsease
Genome-wide Replication And Meta-analysis (CARDIoGRAM)
study: a genome-wide association meta-analysis involving more
than 22 000 cases and 60 000 controls. Circ. Cardiovasc. Genet.
2010;3(5):475-483. DOI 10.1161/CIRCGENETICS.109.899443.

Rau C.D., Lusis A.J., Wang Y. Genetics of common forms of heart
failure. Curr. Opin. Cardiol. 2015;30(3):222-227. DOI 10.1097/
HCO.0000000000000160

Refsgaard L., Holst A.G., Sadjadieh G., Haunsø S., Nielsen J.B., Olesen
M.S. High prevalence of genetic variants previously associated
with LQT syndrome in new exome data. Eur. J. Hum. Genet. 2012;
20(8):905-908. DOI 10.1038/ejhg.2012.23.

Reinthaler M., Braune S., Lendlein A., Landmesser U., Jung F. Platelets
and coronary artery disease: interactions with the blood vessel wall
and cardiovascular devices. Biointerphases. 2016;11(2):29702. DOI
10.1116/1.4953246

Rexiati M., Sun M., Guo W. Muscle-specific mis-splicing and heart
disease exemplified by RBM20. Genes. 2018;9(1):18. DOI 10.3390/
genes9010018.

Rifkind J.M., Mohanty J.G., Nagababu E., Salgado M.T., Cao Z. Potential
modulation of vascular function by nitric oxide and reactive oxygen
species released from erythrocytes. Front. Physiol. 2018;9:690.
DOI 10.3389/fphys.2018.00690.

Rodríguez-Fdez S., Bustelo X.R. The Vav GEF family: an evolutionary
and functional perspective. Cells. 2019;8(5):465. DOI 10.3390/
cells8050465.

Roman T.S., Mohlke K.L. Functional genomics and assays of regulatory
activity detect mechanisms at loci for lipid traits and coronary artery
disease. Curr. Opin. Genet. Dev. 2018;50:52-59. DOI 10.1016/
j.gde.2018.02.004.

Roth G.A., Johnson C., Abajobir A., Abd-Allah F., Abera S.F., Abyu G.,
Ahmed M., Aksut B., Alam T., Alam K., … Yip P., Yonemoto N.,
Younis M., Yu C., Vos T., Naghavi M., Murray C. Global, regional,
and national burden of cardiovascular diseases for 10 causes, 1990
to 2015. J. Am. Coll. Cardiol. 2017;70:1-25. DOI 10.1016/j.jacc.
2017.04.052.

Samman Tahhan A., Hammadah M., Sandesara P.B., Hayek S.S.,
Kalogeropoulos A.P., Alkhoder A., Mohamed Kelli H., Topel M.,
Ghasemzadeh N., Chivukula K., Ko Y.-A., Aida H., Hesaroieh I.,
Mahar E., Kim J.H., Wilson P., Shaw L., Vaccarino V., Waller E.K.,
Quyyumi A.A. Progenitor cells and clinical outcomes in patients
with heart failure. Circ. Heart. Fail. 2017;10(8):e004106. DOI
10.1161/CIRCHEARTFAILURE.117.004106.

Schunkert H., von Scheidt M., Kessler T., Stiller B., Zeng L., Vilne B.
Genetics of coronary artery disease in the light of genome-wide association
studies. Clin. Res. Cardiol. 2018;107(Suppl.2):2-9. DOI
652 10.1007/s00392-018-1324-1.

Seidelmann S.B., Smith E., Subrahmanyan L., Dykas D., Abou
Ziki M.D., Azari B., Hannah-Shmouni F., Jiang Y., Akar J.G.,
Marieb M., Jacoby D., Bale A.E., Lifton R.P., Mani A. Application
of whole exome sequencing in the clinical diagnosis and management
of inherited cardiovascular diseases in adults. Circ. Cardiovasc.
Genet. 2017;10(1):e001573. DOI 10.1161/CIRCGENETICS.
116.001573

Shukla S.K., Rafiq K. Proteasome biology and therapeutics in cardiac
diseases. Transl. Res. 2019;205:64-76. DOI 10.1016/j.trsl.2018.
09.003.

Siebring-van Olst E., Blijlevens M., de Menezes R.X., van der Meulen-
Muileman I.H., Smit E.F., van Beusechem V.W. A genome-wide
siRNA screen for regulators of tumor suppressor p53 activity in human
non-small cell lung cancer cells identifies components of the
RNA splicing machinery as targets for anticancer treatment. Mol.
Oncol. 2017;11(5):534-551. DOI 10.1002/1878-0261.12052

Sloan A., Gona P., Johnson A.D. Cardiovascular correlates of platelet
count and volume in the Framingham Heart Study. Ann. Epidemiol.
2015;25(7):492-498. DOI 10.1016/j.annepidem.2015.01.010.

Smith A.J.P., Deloukas P., Munroe P.B. Emerging applications of genome-
editing technology to examine functionality of GWAS-associated
variants for complex traits. Physiol. Genomics. 2018;50(7):
510-522. DOI 10.1152/physiolgenomics.00028.2018.

Smith J.G., Newton-Cheh C. Genome-wide association studies of lateonset
cardiovascular disease. J. Mol. Cell. Cardiol. 2015;83:131-
141. DOI 10.1016/j.yjmcc.2015.04.004.

Suske G. NF-Y and SP transcription factors – new insights in a longstanding
liaison. Biochim. Biophys. Acta Gene Regul. Mech. 2017;
1860(5):590-597. DOI 10.1016/j.bbagrm.2016.08.011

Szklarczyk D., Gable A.L., Lyon D., Junge A., Wyder S., Huerta-Cepas
J., Simonovic M., Doncheva N.T., Morris J.H., Bork P., Jensen
L.J., von Mering C. STRING v11: protein–protein association
networks with increased coverage, supporting functional discovery
in genome-wide experimental datasets. Nucleic Acids Res. 2019;
47(D1):D607-D613. DOI 10.1093/nar/gky1131.

Uysal H.B., Dağlı B., Akgüllü C., Avcil M., Zencir C., Ayhan M., Sönmez
H.M. Blood count parameters can predict the severity of coronary artery disease. Korean J. Intern. Med. 2016;31(6):1093-1100.
DOI 10.3904/kjim.2015.199

Vagapova E.R., Spirin P.V., Lebedev T.D., Prassolov V.S. The role of
TAL1 in hematopoiesis and leukemogenesis. Acta Naturae. 2018;
10(1):15-23.

van den Hoogenhof M.M.G., Pinto Y.M., Creemers E.E. RNA splicing.
Circ. Res. 2016;118:454. DOI 10.1161/CIRCRESAHA.115.
307872.

Vélez P., García Á. Platelet proteomics in cardiovascular diseases.
Transl. Proteomics. 2015;7:15-29. DOI 10.1016/j.trprot.2014.09.
002.

Völkel S., Stielow B., Finkernagel F., Berger D., Stiewe T., Nist A.,
Suske G. Transcription factor Sp2 potentiates binding of the TALE
homeoproteins Pbx1:Prep1 and the histone-fold domain protein
Nf-y to composite genomic sites. J. Biol. Chem. 2018;293(50):
19250-19262. DOI 10.1074/jbc.RA118.005341.

Wallace E., Howard L., Liu M., O’Brien T., Ward D., Shen S., Prendiville
T. Long QT syndrome: genetics and future perspective. Pediatr.
Cardiol. 2019;40(7):1419-1430. DOI 10.1007/s00246-019-02151-x.

Wan G., Ji L., Xia W., Cheng L., Zhang Y. Screening genes associated
with elevated neutrophil-to-lymphocyte ratio in chronic heart
failure. Mol. Med. Rep. 2018;18(2):1415-1422. DOI 10.3892/mmr.
2018.9132.

Wang M., Tai C., E W., Wei L. DeFine: deep convolutional neural networks
accurately quantify intensities of transcription factor-DNA
binding and facilitate evaluation of functional non-coding variants.
Nucleic Acids Res. 2018;46(11):e69-e69. DOI 10.1093/nar/gky215.

Wang Y., Wang J.-G. Genome-wide association studies of hypertension
and several other cardiovascular diseases. Pulse. 2018;6(3-4):169-
186. DOI 10.1159/000496150

Ward L.D., Kellis M. Interpreting noncoding genetic variation in complex
traits and human disease. Nat. Biotechnol. 2012;30(11):1095-
1106. DOI 10.1038/nbt.2422.

Wijers M., Rimbert A., Dalila N., Fedoseienko A., Wolters K., Dekker
D., Smit M., Levels H., Huijkman N., Kloosterhuis N., Hofker
M., Billadeau D., van Deursen J., Horton J., Burstein E., Tybjaerg-
Hansen A., Kuivenhoven J.A., van de Sluis B. A regulatory
role of the endosomal sorting machinery in controlling plasma LDL
cholesterol levels and atherosclerosis in mice and humans. Atheroscler.
Suppl. 2018;32:18. DOI 10.1016/j.atherosclerosissup.2018.
04.053.

Yang J., Gonon A.T., Sjoquist P.-O., Lundberg J.O., Pernow J. Arginase
regulates red blood cell nitric oxide synthase and export of cardioprotective
nitric oxide bioactivity. Proc. Natl. Acad. Sci. USA. 2013;
110(37):15049-15054. DOI 10.1073/pnas.1307058110.

Yang J., Villar V.A.M., Rozyyev S., Jose P.A., Zeng C. The emerging
role of sorting nexins in cardiovascular diseases. Clin. Sci. 2019;
133(5):723-737. DOI 10.1042/CS20190034.

Zhang L., Xue G., Liu J., Li Q., Wang Y. Revealing transcription factor
and histone modification co-localization and dynamics across
cell lines by integrating ChIP-seq and RNA-seq data. BMC Genom.
2018;19(Suppl.10):914. DOI 10.1186/s12864-018-5278-5.

Zhong M., Komarova Y., Rehman J., Malik A.B. Mechanosensing
Piezo channels in tissue homeostasis including their role in lungs.
Pulm. Circ. 2018;8(2):1-6. DOI 10.1177/2045894018767393.

Zhou Z., Mahdi A., Tratsiakovich Y., Zahorán S., Kövamees O., Nordin
F., Uribe Gonzalez A.E., Alvarsson M., Östenson C.-G., Andersson
D.C., Hedin U., Hermesz E., Lundberg J.O., Yang J., Pernow J.
Erythrocytes from patients with type 2 diabetes induce endothelial
dysfunction via arginase I. J. Am. Coll. Cardiol. 2018;72(7):769-
780. DOI 10.1016/j.jacc.2018.05.052.

